# Dynamin‐like protein 1 cleavage by calpain in Alzheimer’s disease

**DOI:** 10.1111/acel.12912

**Published:** 2019-02-14

**Authors:** Sirui Jiang, Changjuan Shao, Fangqiang Tang, Wenzhang Wang, Xiongwei Zhu

**Affiliations:** ^1^ Department of Pathology Case Western Reserve University Cleveland Ohio

**Keywords:** Alzheimer’s disease, amyloid‐β, calpain, DLP1/Drp1, mitochondrial distribution, mitochondrial dynamics

## Abstract

Abnormal mitochondrial dynamics contributes to mitochondrial dysfunction in Alzheimer's disease (AD), yet the underlying mechanism remains elusive. In the current study, we reported that DLP1, the key mitochondrial fission GTPase, is a substrate of calpain which produced specific N‐terminal DLP1 cleavage fragments. In addition, various AD‐related insults such as exposure to glutamate, soluble amyloid‐β oligomers, or reagents inducing tau hyperphosphorylation (i.e., okadaic acid) led to calpain‐dependent cleavage of DLP1 in primary cortical neurons. DLP1 cleavage fragments were found in cortical neurons of CRND8 APP transgenic mice which can be inhibited by calpeptin, a potent small molecule inhibitor of calpain. Importantly, these N‐terminal DLP1 fragments were also present in the human brains, and the levels of both full‐length and N‐terminal fragments of DLP1 and the full‐length and calpain‐specific cleavage product of spectrin were significantly reduced in AD brains along with significantly increased calpain. These results suggest that calpain‐dependent cleavage is at least one of the posttranscriptional mechanisms that contribute to the dysregulation of mitochondrial dynamics in AD.

## 1 INTRODUCTION

Alzheimer's disease (AD), the most common cause of dementia in the elderly, is characterized by progressive neurodegeneration and cognitive impairment. The pathological hallmarks of AD include accumulation of extracellular plaques composed of amyloid‐β (Aβ) and intracellular neurofibrillary tangles composed of hyperphosphorylated tau in the hippocampus and cortex of the human brain (Mattson, 2004). There is currently no cure or viable treatment for the neurodegeneration and progressive dementia in AD. According to the World Alzheimer's Report, there are an ever‐increasing number of people living with AD and will increase to 131.5 million by 2050.

Although the exact pathogenic mechanism of AD remains elusive, much research has been done to implicate mitochondrial dysfunction as an early prominent feature in susceptible neurons that plays a critical role in the pathogenesis of the disease (Swerdlow, 2016; Wang et al., 2014). Such a primary role is underscored by the fact that defective glucose utilization and energy metabolism is a well‐documented abnormality preceding functional impairment in patients with mild cognitive impairment, a prodromal stage of AD, and in AD (Swerdlow, 2016). While mechanisms underlying mitochondrial dysfunction in AD remain incompletely understood, recent studies from multiple groups demonstrated that abnormal mitochondrial dynamics and distribution are likely involved: overexpression of familial AD APP mutations or exposure to soluble Aβ oligomers induced profound mitochondrial fragmentation, ultrastructural damage, and reduced mitochondrial distribution in neuronal processes in neuronal culture (Du et al., 2010; Manczak et al., 2010; Wang et al., 2009; Wang, Su, Siedlak, et al., 2008). These deficits are causally involved in Aβ‐induced mitochondrial dysfunction and synaptic abnormalities in primary hippocampal or cortical neurons in vitro (Wang, Su, Fujioka, & Zhu, 2008). Mitochondrial damage in the form of irregular distribution and round and engorged mitochondria are also documented in AD mouse models (Trushina et al., 2012; Wang et al., 2017). Mitochondrial numbers were reduced and became dystrophic and fragmented in the vicinity of plaques in APP/PS1 mice revealed by real‐time imaging study (Xie et al., 2013). Similarly, swollen mitochondria with extensive ultrastructural damage and abnormal distribution are also observed in the brain of AD patients (Hirai et al., 2001; Wang et al., 2009).

Mitochondria are dynamic organelles that undergo fusion and fission controlled by large GTPases: Mitochondrial fission is regulated by cytosolic protein dynamin‐like protein 1 (DLP1) which translocates to mitochondrial outer membrane during fission with the assistance of mitochondrial outer membrane proteins such as Fis1 or Mff1 (Mishra & Chan, 2014). Mitochondrial fusion is regulated by mitofusin 1 and 2 (MFN1/2) on the outer mitochondrial membrane and OPA1 on the inner mitochondrial membrane (Mishra & Chan, 2014). Mitochondrial dynamics is critical for maintaining the homeostasis of mitochondria including the tight regulation of their morphology and distribution according to the metabolic need of the cells. Changes in mitochondrial dynamics significantly impact almost all aspects of mitochondrial function, and defects in the large GTPases involved in mitochondrial fission/fusion cause human neurological diseases. Interestingly, our prior studies revealed that all these large GTPase involved in mitochondrial fission (DLP1) and fusion (Mfn1/2 and OPA1) are decreased in the fibroblasts and brain of AD patients and in soluble Aβ oligomer‐treated cells (Wang, Su, Fujioka, et al., 2008; Wang et al., 2009). It appears that these changes not only result in mitochondrial fragmentation, but also lead to mitochondrial abnormal distribution since DLP1 overexpression could rescue Aβ‐induced depletion of mitochondria in the neuronal processes (Wang, Su, Fujioka, et al., 2008). Our results were consistent with a prior study where DLP1 knockdown led to depletion of mitochondria in the dendrites and its overexpression helps to repopulate dendrites and synapse with mitochondria (Li, Okamoto, Hayashi, & Sheng, 2004). Reduced levels of fusion GTPases in AD brain were consistently replicated by other groups (Kandimalla & Reddy, 2016; Manczak, Calkins, & Reddy, 2011), and our recent study demonstrated that Mfn2 ablation caused neurodegeneration and other AD‐related deficits in the hippocampus and cortex (Jiang et al., 2018). However, there were inconsistent reports on the levels of DLP1, the fission GTPase, in AD: Several groups confirmed our results of reduced DLP1 in AD brain or fibroblasts (Bossy et al., 2010; Wang, Song, Tan, Albers, & Jia, 2012) but other group demonstrated increased levels of DLP1 in AD brain (Kandimalla & Reddy, 2016; Reddy et al., 2011). While it remains to resolve how DLP1 is changed in AD brain, it was demonstrated that DLP1 interacts with tau and/or Aβ (Manczak et al., 2011; Manczak & Reddy, 2012), which likely plays a critical role in mitochondrial fragmentation observed in AD.

To understand the mechanism(s) underlying the changes in the expression of these fission/fusion GTPases, we investigated the potential involvement of aberrant calcium signaling since calcium dyshomeostasis has been well documented in AD as many of the clinical mutations in the presenilin (PS1/PS2) genes have been shown to disrupt the calcium signaling cascade (LaFerla, 2002). The aberrant calcium signaling leads to excitotoxicity and may be a mechanism of neuronal death in AD. It is well established that Aβ oligomers induce a rapid and sustained increase in intracellular calcium in neurons and Aβ induces neuronal abnormalities including spine loss and synaptic dysfunction likely through the activation of calcium‐dependent signaling molecules such as calpain (Li et al., 2011). Interestingly, there is evidence that calpain activation leads to cleavage of the dynamin protein in an AD cell model (Kelly, Vassar, & Ferreira, 2005). Given the similarity between dynamin and dynamin‐related proteins involved in mitochondrial fission and fusion, we hypothesized that calpain activation may be involved in the reduction of these mitochondrial fission/fusion proteins in AD. Therefore, in this study we aimed to investigate the posttranscriptional regulation of mitochondrial fission/fusion GTPases with a focus on the effects of calpain activation and reduced levels of DLP1, the key protein involved in mitochondrial fission and distribution. We observed the cleavage of DLP1 by calpain into several fragments at ~65 kDa and 50 kDa in size. This cleavage can be triggered by AD‐relevant insults such as exposure to glutamate, soluble Aβ oligomers, or agents inducing tau phosphorylation. Cleavage fragments of DLP1 as well as the cytoskeletal protein, spectrin, along with increased active calpain are observed in AD patient brains supporting our theory that calpain is a protease that plays a role in the reduction of mitochondrial proteins seen in AD.

## 2 RESULTS

### 2.1 Cleavage of recombinant DLP1 by calpain

**Figure 1:**
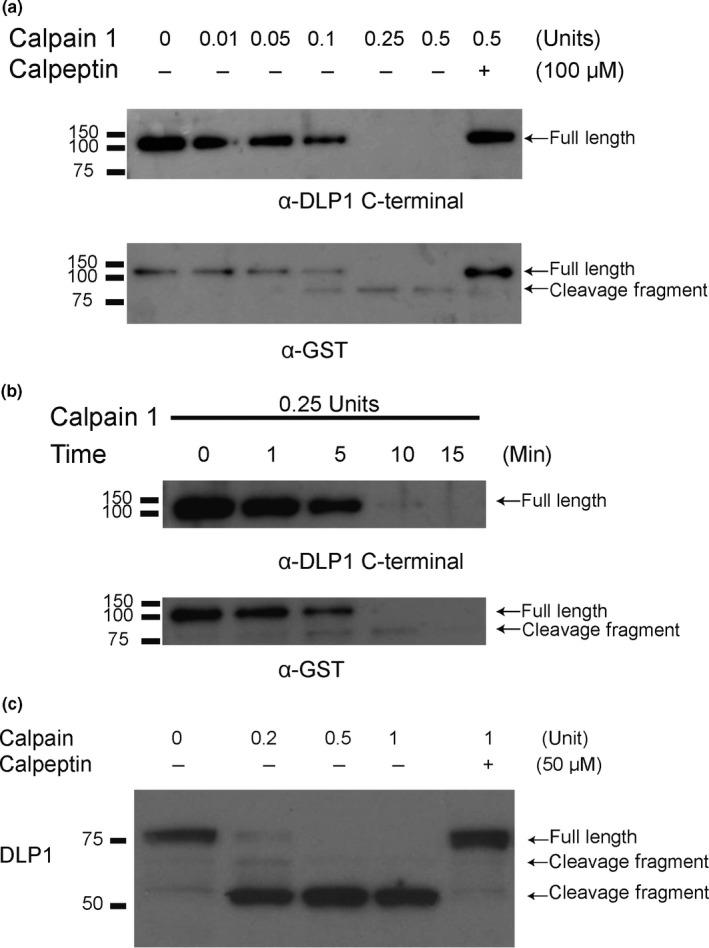
Dose‐ and time‐dependent cleavage of recombinant DLP1 tagged with GST at N‐terminal by calpain‐1 and DLP1 cleavage by calpain in M17 neuroblastoma cells. (a) 160 ng recombinant GST‐DLP1 was incubated with calpain‐1 at 30°C at the indicated concentration for 30 min in the absence/presence of calpeptin, and the remaining GST‐DLP1 was visualized by Western Blotting with a C‐terminal specific DLP1 antibody (upper panel) or a GST antibody (lower panel). (b) 160 ng recombinant GST‐DLP1 was incubated with 0.25 units calpain‐1 at 30°C for the indicated duration, and the remaining GST‐DLP1 was visualized by Western Blotting with a C‐terminal specific DLP1 antibody (upper panel) or a GST antibody (lower panel). (c) Representative Western Blot of DLP1 N‐terminal specific antibody (i.e., DLP1 D6C7) in whole cell lysates from M17 cells incubated with calpain‐1 at 30°C at the indicated concentrations for 30 min in the absence/presence of calpeptin. Similar data were obtained with three independent experiments

To investigate whether DLP1 is directly cleaved by calpain, we incubated recombinant N‐terminal GST‐tagged DLP1 with calpain‐1 in reaction buffer for various times and concentrations and examined the DLP1 levels by Western Blot analysis. Using a C‐terminal DLP1 antibody (i.e., DLP1 C5 antibody against amino acids 560–736 region of DLP1), a significant reduction in the level of full‐length GST‐DLP1 is observed with 0.05 units (0.05 U) of calpain‐1 after a 30‐min treatment, which became more significantly reduced with higher units of calpain‐1 (Figure 1a). In fact, 30‐min treatment with 0.25 or 0.5 units of calpain‐1 resulted in total loss of full‐length GST‐DLP1, reflecting a dose‐dependent effect. We also used a GST antibody to detect GST‐DLP1 after cleavage by calpain. Interestingly, in addition to the dose‐dependent reduction in the level of full‐length GST‐DLP1, we also observed the appearance of a specific band around 75 kDa with the 30‐min treatment of 0.05 units of calpain‐1 which peaked with the treatment of 0.25 units of calpain‐1 but then decreased with the treatment of 0.5 units of calpain‐1. Since the DLP1 was N‐terminally tagged with GST, this ~75 kDa band likely reflects a ~50 kDa N‐terminal cleavage fragment of DLP1. The reduction of full‐length GST‐DLP1 and appearance of cleavage fragments were completely prevented when calpeptin, a specific calpain inhibitor, was present along with calpain, demonstrating the specificity of the calpain cleavage reaction. Treatment with 0.25 units of calpain‐1 led to gradual reduction of full‐length GST‐DLP1 with time until its full cleavage after 10 min as revealed by the C‐terminal DLP1 antibody (Figure 1b). Similarly, we also observed the appearance of the 75 kDa fragment as early as 1 min which peaked at 10 min and was completely gone at 15 min (Figure 1b).

To further investigate this calpain‐dependent cleavage of DLP1, whole cell lysate from M17 neuroblastoma cells was incubated with calpain‐1 at varying concentrations for 30 min. Using an antibody against N‐terminal DLP1 (i.e., DLP1 D6C7 antibody), we confirmed the dose‐dependent reduction of full‐length DLP1 by calpain‐1 (Figure 1c). Importantly, accompanying the reduced full‐length DLP1, there was an accumulation of a major band around 50 kDa after calpain‐1 treatment. In fact, the accumulation of this band even persisted with up to 4 hr of 1 unit calpain‐1 treatment (not shown). There was also a weak band around 65 kDa in the lysate treated with 0.2 units calpain which was gone in the lysate treated with 0.5 units calpain, likely reflecting a transient intermediate DLP1 fragment generated by calpain cleavage. In fact, these two cleavage products were also present in the M17 lysate at basal condition as faint bands, suggesting such cleavage likely occurs endogenously. The concurrent treatment with calpeptin abolished both the reduction of full‐length DLP1 and the appearance/accumulation of DLP1 N‐terminal fragments. These results suggest that DLP1 is a substrate of calpain and that calpain cleavage of DLP1 yields N‐terminal fragments of 65 and 50 kDa in size. We therefore used DLP1 N‐terminal antibody to detect these calpain‐dependent N‐terminal DLP1 fragments in later studies.

### 2.2 DLP1 cleavage by calpain in glutamate‐treated neurons

**Figure 2:**
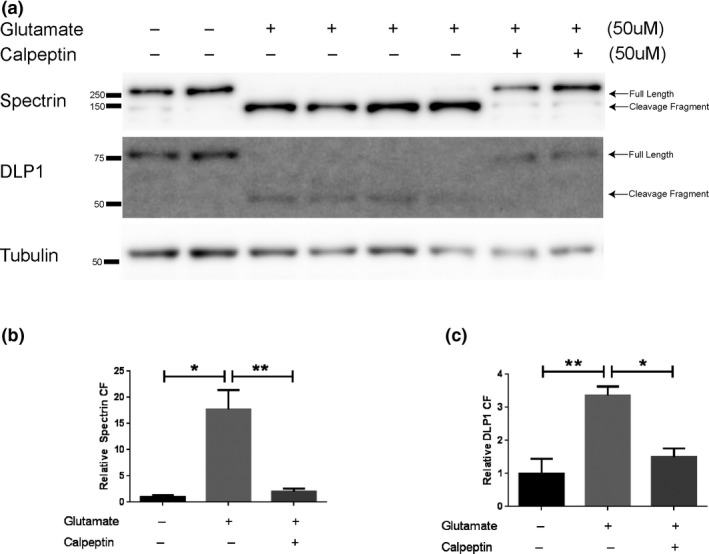
Calpain‐dependent cleavage of spectrin and DLP1 in glutamate‐treated rat primary cortical neurons. (a) Representative Western Blots of DLP1 in primary cortical neurons (DIV12) treated with 50 µM glutamate in the absence/presence of 50 µM calpeptin for 4 hr. Spectrin was probed as a positive control for calpain activation; β‐tubulin was probed as an internal loading control. (b, c) Quantitative analysis of levels of 150 kDa cleavage fragment (CF) of spectrin CF (b) and 50 kDa cleavage fragment (CF) of DLP1 (c). The expression levels of all proteins were normalized to tubulin and expressed as a relative to nontreated control values of each respective protein. Data are presented as the mean ± *SEM* of three independent experiments (*p < 0.05, **p < 0.001)

To investigate calpain‐dependent DLP1 cleavage in cells, we examined the DLP1 protein levels in rat primary neurons treated with glutamate, which induces calcium dyshomeostasis and excitotoxicity through the activation of the NMDA and AMPA receptors and is believed to be involved in AD. Under this condition, glutamate treatment led to activation of calpain activity as revealed by the almost complete loss of 250 kDa full‐length spectrin protein and the appearance of a major 150 kDa spectrin cleavage product which is specific to calpain activity (Figure 2a,b). Similarly, the full‐length DLP1 protein was almost completely gone in glutamate‐treated cells which was accompanied by an accumulation of the 50 kDa N‐terminal fragment (Figure 2a,c) as was identified in both the cell free cleavage assay and M17 cell lysate (Figure 1). With the addition of calpeptin to the glutamate treatments, the loss of full‐length spectrin and DLP1 along with the increase in the specific cleavage fragments of these two proteins was significantly inhibited (Figure 2a–c).

### 2.3 DLP1 cleavage by calpain in Aβ‐treated neurons

**Figure 3:**
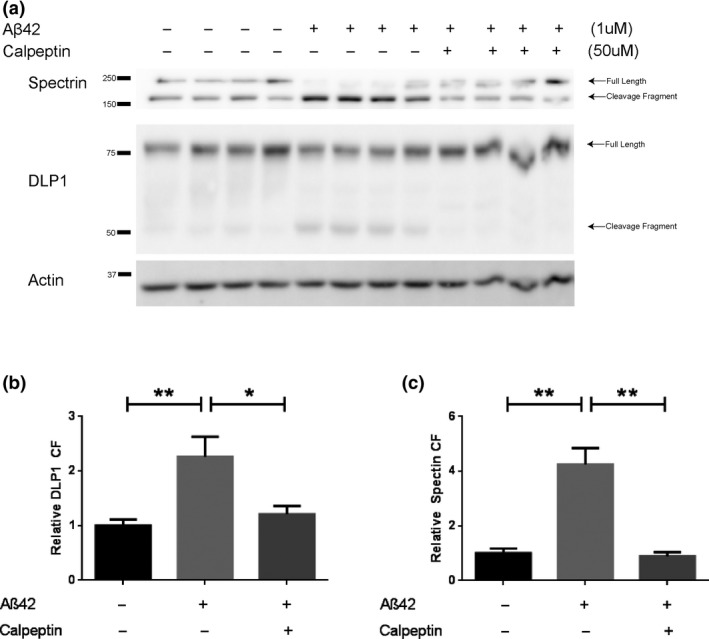
Calpain‐dependent cleavage of spectrin and DLP1 in rat primary cortical neurons treated with soluble Aβ oligomers. (a) Representative Western Blots of DLP1 in primary cortical neurons (DIV12) treated with 1 µM soluble Aβ oligomers in the absence/presence of 50 µM calpeptin for 4 hr. Spectrin was probed as a positive control for calpain activation; actin was probed as an internal loading control. (b, c) Quantitative analysis of levels of 150 kDa cleavage fragment (CF) of spectrin (c) and 50 kDa cleavage fragment (CF) of DLP1 (b). The protein levels were normalized to actin and expressed as a relative to nontreated control values of each respective protein. Data are presented as the mean ± *SEM* of three independent experiments (*p < 0.05, **p < 0.001)

To investigate a potential mechanism underlying DLP1 reduction in AD, we examined DLP1 levels in rat primary neurons treated with soluble Aβ oligomers. As expected, treatment with Aβ oligomers led to decreased full‐length spectrin and a significant increase in the 150 kDa spectrin cleavage fragment (Figure 3a,c), indicating that treatment with Aβ caused calpain activation. Similarly, a significant increase in the 50 kDa cleavage fragment was revealed when detected by the DLP1 N‐terminal antibody (Figure 3a,b). Co‐treatment with calpeptin in Aβ‐treated neurons prevented the activation of calpain as demonstrated by the restoration of full‐length spectrin along with the reduction of 150 kDa cleavage fragment to the level similar to vehicle‐treated cells (Figure 3a,c). Under this condition, the appearance of DLP1 cleavage fragment was also completely reversed (Figure 3a,b).

### 2.4 DLP1 cleavage by calpain in okadaic acid‐treated neurons

**Figure 4:**
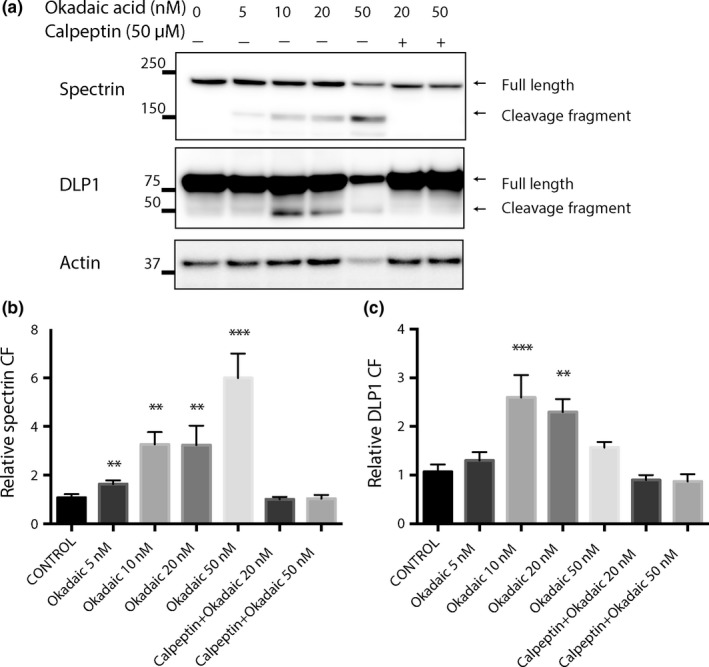
Calpain‐dependent cleavage of spectrin and DLP1 in rat primary cortical neurons treated with okadaic acid. (a) Representative Western Blots of DLP1 in primary cortical neurons (DIV12) treated with okadaic acids at the indicated concentration in the absence/presence of 50 µM calpeptin for 24 hr. Spectrin was probed as a positive control for calpain activation; actin was probed as an internal loading control. (b, c) Quantitative analysis of levels of 150 kDa cleavage fragment (CF) of spectrin (b) and 50 kDa cleavage fragment (CF) of DLP1 (c). The protein levels were normalized to actin and expressed as a relative to nontreated control values of each respective protein. Data are presented as the mean ± *SEM* of three independent experiments. Data are presented as the mean ± *SEM* (**p < 0.01, ***p < 0.001)

Hyperphosphorylation of tau protein is also involved in the pathogenesis of AD. We further analyzed the impact of phosphorylated tau on calpain cleavage of DLP1. To mimic the neurotoxicity of phosphorylated tau in cell cultures, we treated primary rat neurons with okadaic acid which is an inhibitor of serine/threonine phosphatases 1 and 2A and induces hyperphosphorylation of tau (Kamat, Rai, & Nath, 2013). Interestingly, okadaic acid treatment induced dose‐dependent degradation of both full‐length spectrin and DLP1 proteins in cultured neurons and the appearance of calpain cleavage products of 150 kDa spectrin fragment and 50 kDa DLP1 fragment (Figure 4a‐c). As a specificity control, concurrent calpeptin treatment abolished the cleavage of both spectrin and DLP1 after okadaic acid treatment (Figure 4b,c).

### 2.5 DLP1 cleavage by calpain is not a general response during mitochondrial fragmentation

It is known that a variety of stresses that disrupt mitochondrial function also cause mitochondrial fragmentation (De Vos, Allan, Grierson, & Sheetz, 2005). To investigate whether calpain‐dependent DLP1 is a general response during mitochondrial fragmentation when mitochondrial function is disrupted, we treated rat primary cortical neurons with rotenone (10–100 nM), a specific inhibitor of complex I, and FCCP (0.5–2 µM), a widely used mitochondrial protonophore that uncouples electron transport and oxidative phosphorylation, at a range of concentrations that disrupt mitochondrial function and cause mitochondrial fragmentation (Barsoum et al., 2006; De Vos et al., 2005) and investigated the calpain‐dependent cleavage of spectrin and DLP1 by Western Blot analysis. Our results indicated that neither rotenone nor FCCP caused any changes in the 150 k Da calpain‐dependent cleavage product of spectrin or increase in the 50 kDa cleavage product of DLP1 (Supporting Information Figure S1), suggesting that DLP1 cleavage by calpain is not a general response during mitochondrial fragmentation.

### 2.6 Calpain‐dependent cleavage of DLP1 in neurons of APP transgenic mouse model

**Figure 5:**
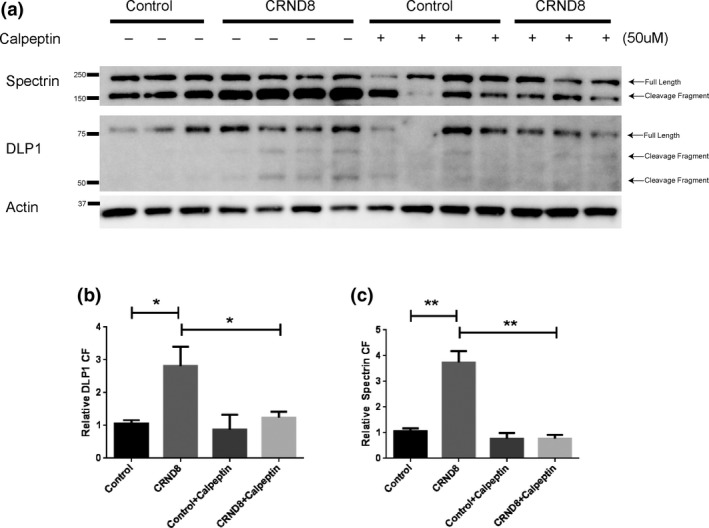
Calpain‐dependent cleavage of spectrin and DLP1 in primary cortical neurons isolated from CRND8 APP transgenic mice. (a) Representative Western Blots of DLP1 in primary cortical neurons (DIV12) isolated from either CRND8 mice or littermate control mice treated with/without 50 µM calpeptin for 4 hr. Spectrin was probed as a positive control for calpain activation; actin was probed as an internal loading control. (b, c) Quantitative analysis of levels of 150 kDa cleavage fragment (CF) of spectrin (c) and 50 kDa cleavage fragment (CF) of DLP1 (b). The protein levels were normalized to actin and expressed as a relative to nontreated control values of each respective protein. Data are presented as the mean ± *SEM* of three independent experiments (*p < 0.05, **p < 0.001)

We next investigated whether the calpain‐dependent cleavage of DLP1 is present in neurons of CRND8 mice, a widely used APP transgenic (Tg) mouse model expressing the APP Swedish (KM670/671NL) and APP Indiana (V717F) mutations that shows early signs of memory impairment as well as striking amyloid plaque pathology (Yin et al., 2018). In the primary cortical neurons isolated from CRND8 mice, we observed significant presence of the 150 kDa calpain‐dependent cleavage fragment of spectrin compared to neurons from their nonTg littermate controls (Figure 5a,c), suggesting a higher calpain activity in the CRND8 neurons. In conjunction with activated calpain, we also observed a significant presence of the 65 and 50 kDa cleavage fragments of DLP1 (Figure 5a,b). Furthermore, treatment with calpeptin inhibited the increased levels of 150 kDa cleavage fragment of spectrin as well as the 65 and 50 kDa DLP1 cleavage fragments (Figure 5a‐c).

### 2.7 DLP1 cleavage in AD brains

**Figure 6:**
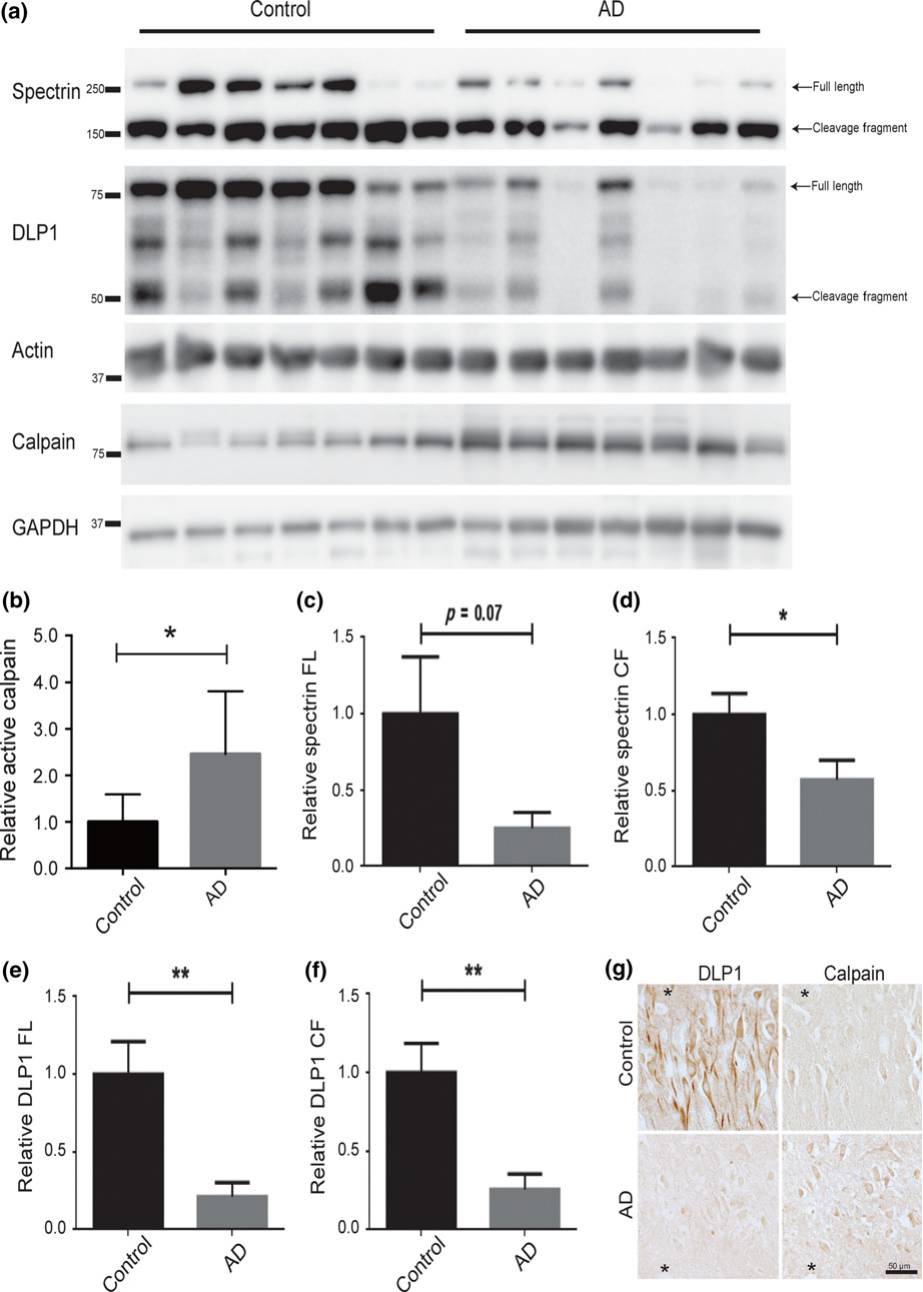
Decreased level of DLP1 in Alzheimer's disease (AD) brain. (a) Expression of DLP1 and spectrin in AD and age‐matched control brains was analyzed by Western Blotting with antibodies to calpain, spectrin, and DLP1. Calpain antibody only recognizes a specific band around 76 kDa corresponding to active calpain. GAPDH or actin blot was used as internal loading control. (b–e) Quantitative analysis of levels of 76 kDa active calpain (b), full‐length (c), or 150 kDa cleavage fragment (d) of spectrin and full‐length (e) or 50 kDa cleavage fragment (f) of DLP1. The protein levels were normalized to GAPDH or actin and expressed as a relative to control values of each respective protein. Data are presented as the mean ± *SEM* of three independent experiments (*p < 0.05, **p < 0.001). (g) Representative immunocytochemistry of DLP1 and calpain in the hippocampus in adjacent serial sections of AD (*N* = 6) and control cases (*N* = 5) revealed reduced neuronal DLP1 immunoreactivity associated with increased neuronal calpain immunoreactivity in AD hippocampus compared to age‐matched control cases. *The landmark vessel in adjacent sections of AD or control

Our prior studies demonstrated that protein levels of DLP1 are significantly reduced but mRNA levels of DLP1 are not changed in the brain of human AD patients compared to age‐matched control patients (Wang et al., 2009), suggesting the involvement of post‐transcriptional regulation of DLP1 expression. To investigate whether calpain activation is involved in the reduced expression of DLP1 in AD brain, we first investigated the levels and activity of calpain in brain homogenates from AD and age‐matched control patients by performing Western Blot analysis of calpain and calpain‐dependent cleavage products of spectrin. As an 80 kDa protease, calpain is activated by its autoproteolytic cleavage into 76‐ and 58‐kDa fragments. As reported previously (Atherton et al., 2014), the calpain antibody used in this study only recognized a single prominent band around 76 kDa in both AD and control brain homogenates, corresponding to its active form. Quantification analysis revealed that the level of this active form of calpain is significantly increased in AD brain (Figure 6a,b). Both the full‐length and calpain‐dependent 150 kDa cleavage fragments of spectrin were present in both AD and control samples. There was a trend (*p* = 0.07) of decrease in the level of full‐length spectrin and a significant decrease in the level of 150 kDa cleavage product in the brain tissues from AD patients as compared to that from the age‐matched nonAD control patients (Figure 6a,c,d). We also probed the DLP1 cleavage products in these brain homogenates. Similarly, our N‐terminal DLP1 antibody revealed that both full‐length and the two cleavage fragments (i.e., 65 and 50 kDa bands) of DLP1 were also present in AD and control sample (Figure 6a). Consistent with our previous report (Wang et al., 2009), there was a significant decrease in the level of full‐length DLP1 (Figure 6e). Moreover, the levels of the cleavage fragments of DLP1 decreased in the AD samples as compared to the age‐matched nonAD controls samples (Figure 6f). Relative levels of DLP1 and calpain were also compared in hippocampal neurons in adjacent serial sections of AD and control cases using immunocytochemistry (Figure 6g). Neuronal staining for DLP1 was highest in the control cases and very weak staining was seen in the AD cases (Figure 6g). Conversely, very little calpain was detected in the same neurons in the control cases, while the AD cases had more neurons with calpain staining, and at higher levels than was found in the controls (Figure 6g).

## 3 DISCUSSION

In this study, we investigated the association between DLP1 levels and calpain activation in the context of AD models and AD brain. By employing the in vitro calpain cleavage assay using GST‐DLP1 recombinant protein and cell lysate from M17 neuroblastoma cells, we demonstrated that DLP1 is indeed a substrate of calpain which produced specific N‐terminal DLP1 fragments. Furthermore, various AD‐related insults such as exposure to glutamate, soluble Aβ oligomers, or reagents inducing tau hyperphosphorylation (i.e., okadaic acid) led to DLP1 cleavage accompanied by calpain activation in primary cortical neurons. DLP1 cleavage fragments were also present in cortical neurons of CRND8 APP transgenic mice. Importantly, calpeptin, a potent small molecule inhibitor of calpain, prevented DLP1 cleavage in all these models, demonstrating the specific involvement of calpain activation in DLP1 cleavage in these models. Lastly, we found the presence of N‐terminal DLP1 fragments in the human brains and the levels of both full‐length and N‐terminal fragments of DLP1 were significantly reduced in AD brains. These results suggest that calpain activation is at least one of the posttranscriptional mechanisms that contribute to the changes in the levels of large GTPases involved in mitochondrial fission/fusion and dysregulation of mitochondrial dynamics in AD.

The major finding in this study is that we firmly established DLP1 as a physiological and AD‐relevant pathophysiological substrate of calpain in cells and in the brain. This was first clearly shown by our in vitro calpain cleavage assay demonstrating a dose‐ and time‐dependent cleavage of recombinant GST‐DLP1 by calpain which was inhibited by calpeptin. This in vitro assay also suggested the presence of an intermediate 50 kDa N‐terminal DLP1 cleavage fragment before its complete digestion by calpain. Indeed, we found this 50 kDa fragment along with another 65 kDa N‐terminal fragment of DLP1 in the M17 cells at basal condition which could be enhanced by calpain treatment and inhibited by calpeptin, suggesting that they are present in the physiological condition by calpain cleavage. These two specific N‐terminal fragments of DLP1 were also present in the brains from elderly human control patients which confirms the physiological presence of these cleavages in vivo. Importantly, the current study demonstrated that these calpeptin‐sensitive cleavages of DLP1 were significantly enhanced by AD‐relevant insults such as treatment with glutamate, soluble Aβ oligomers or okadaic acids, modeling excitotoxicity, Aβ exposure, or tau hyperphosphorylation induction, but not by general insults that disrupt mitochondrial function, which thus demonstrated the involvement of the calpain‐dependent cleavage of DLP1 in AD models. The pathophysiological relevance of calpain‐dependent cleavage of DLP1 is further corroborated by the evidence from cortical neurons of CRND8 APP transgenic mice and brain tissues from AD patients. Calpain activation has been closely linked with AD as it has been shown to cleave: APP that regulates Aβ production (Morales‐Corraliza et al., 2012), tau leading to production of neurotoxic fragments (Ferreira & Bigio, 2011), synaptic proteins like dynamin‐1 (Kelly et al., 2005), and NMDA receptor subunit NR2B important for synaptic health (Simpkins et al., 2003). Our results thus added reduced DLP1 as a victim of enhanced calpain activation in AD which affect mitochondrial dynamics and contribute to mitochondrial dysfunction during AD pathogenesis. Calpain inhibition has been pursued as a therapeutic target for AD (Medeiros et al., 2012) which therefore could also potentially have positive impact on mitochondrial dynamics. Imbalance in kinase and phosphatase results in tau hyperphosphorylation and plays an important role in the pathogenesis of AD. Fission activity of DLP1 is also regulated by phosphorylation at several sites, and cytosolic calcium signaling could either increase DLP1 phosphorylation at Ser616 by Ca^2+^/calmodulin‐dependent kinase II (Xu et al., 2016) or decreased DLP1 phosphorylation at Ser637 through calcineurin (Cereghetti et al., 2008). While it was demonstrated that DLP1 phosphorylation at Ser616 is increased in AD brain (Wang et al., 2009), how phosphorylation of DP1 at Ser637 is changed in AD is not clear. Furthermore, whether and how the phosphorylation at these sites affects or relates to calpain cleavage of DLP1 remains to be determined.

Previous studies of postmortem AD patient brains show increased calpain activity in end‐stage AD brain (Atherton et al., 2014; Jin et al., 2015; Saito, Elce, Hamos, & Nixon, 1993) as well as elevated levels of cleaved products of several calpain substrates (Atherton et al., 2014; Liu et al., 2005). In the current study, while we also observed increased levels of active calpain in AD brain, we observed clear reduction, sometimes a complete depletion, in the levels of full‐length spectrin or DLP1 in the brains from AD patients, and also found significantly reduced levels of calpain‐specific 150 kDa fragment of spectrin along with reduced levels of two N‐terminal fragments of DLP1. This complete depletion of DLP1 full‐length and N‐terminal fragments in nearly all of the AD samples we examined, in our opinion, implicated excessive calpain activation that resulted in more complete degradation of DLP1 in AD. Along this reasoning, it was demonstrated that calpain activation is associated with disease progression through various Braak stages and the levels of full‐length and calpain‐dependent cleavage products of certain substrates such as CAST peaked at earlier stages but both declined at later stage (Kamat et al., 2013). It is therefore of interest to further determine how the levels of DLP1 and its cleavage fragments change along different stages during the course of AD. Nevertheless, our study does not preclude the potential involvement of other proteases in the cleavage of DLP1. For example, studies have shown that caspases may indirectly regulate cleavage of mitochondrial GTPases such as OPA1 (Loucks et al., 2009) and DLP1 during apoptosis (Estaquier & Arnoult, 2007).

The presence of 65 and 50 kDa DLP1 fragments at physiological conditions, especially in the brain of elderly controls, may be interesting. The function of DLP1 has been thoroughly explored in both yeast and mammals as it is similar to the classic dynamins in membrane scission and remodeling of mitochondria (Sesaki, Adachi, Kageyama, Itoh, & Iijima, 2014). It is a cytosolic protein that forms dimers and tetramers and is recruited to the mitochondrial surface through interactions with various outer mitochondrial proteins such as mitochondrial fission factor (Mff), Fis1, and mitochondrial elongation factor (MIEF1/2; Sesaki et al., 2014). Crystal structures show that DLP1 assembles into spirals which mediates mitochondrial scission (Mears et al., 2011). DLP1 contains four domains: an N‐terminal GTPase domain, middle domain, variable domain, and C‐terminal GTPase effector domain (GED; Otera, Ishihara, & Mihara, 2013). The middle domain is important in the regulation of DLP1 self‐assembly into dimers and tetramers (Frohlich et al., 2013). So it can stand to reason that cleavage of DLP1 somewhere in the middle domain resulting in 50 kDa and 65 kDa fragments as shown in our data could lead to dysfunctional oligomerization of DLP1 and therefore affect its function in regulating mitochondrial fission. While DLP1 knockdown could lead to abnormal mitochondrial distribution, how DLP1 cleavage fragments may affect mitochondrial transport and distribution is not clear. Further studies will be needed to map the specific cleavage sites and their impact on DLP1 functions.

In conclusion, this study established DLP1 as a calpain substrate and suggested that calpain activation could contribute to reduced DLP1 levels and mitochondrial dynamics abnormalities and mitochondrial dysfunction in AD.

## 4 EXPERIMENTAL PROCEDURES

### 4.1 Human brain tissues

Samples were obtained at autopsy and, following approved IRB protocols, were received from the Brain Bank at Case Western Reserve University and from the NIH Neurobiobank in accordance with the institutional bioethics guidelines. The diagnosis of Alzheimer's disease was obtained according the NINCDS‐ADRDA group criteria (McKhann et al., 1984). Brain samples from cases of neuropathologically confirmed AD (*n* = 13, ages 67–90 year [76.5 ± 1.8 year, mean ± *SEM*] and PMI range of 3–26 hr [9.1 ± 2 hr], gender: 8M:5F) and neuropathologically normal cases (*n* = 12, ages 60–92 year [77.5 ± 2.6 year] and PMI range 4–25 hr [mean 13.6 ± 1.7 hr], gender: 5M:7F) were used for Western Blot or immunocytochemical studies.

### 4.2 Primary rat and CRND8 mouse neuronal culture

Primary cultures of both rat and mouse cortical neurons were prepared from the brains of embryonic pups at day 18 and day 16, respectively, as previously reported (Kaech & Banker, 2006; Zhao et al., 2017) with some modification. In brief, the cerebral cortices were dissected from the embryonic brain and dissociated by trypsinization for 10 min at room temperature. The resulting cell suspensions were resuspended in neurobasal medium supplemented with B27 (Gibco‐BRL, Waltham, MA, USA) and penicillin‐streptomycin (Thermo Fisher Scientific, Waltham, MA, USA) and plated onto poly‐d‐lysine (Sigma, St. Louis, MO, USA) coated plastic plates. Neurons were maintained at 37°C in 5% CO_2_ for 12 days prior to chemical treatment. After 12 days in vitro (DIV), the neuron culture medium was replaced with fresh medium containing treatments of Aβ, glutamate, and calpeptin as described in the paper.

### 4.3 Drugs

Glutamate (50 μM; Sigma), calpeptin (50 μM; Tocris Bioscience, Minneapolis, MN, USA), FCCP (0.25–2 μM; Sigma), rotenone (10–100 nM; Sigma), and okadaic acid (5–50 nM; Cayman Chemical, Ann Arbor, MI, USA) were added to neuronal cultures at the indicated final concentrations. Aβ oligomers were prepared as previously described (Song et al., 2014). Briefly, lyophilized Aβ peptides were dissolved in dimethyl sulfoxide, diluted in neurobasal without phenol red (Gibco‐BRL) to a final concentration of 1 μM, and incubated at 4°C for 16 hr. Prepared Aβ oligomers were added to neuronal cultures for the indicated times.

### 4.4 Calpain cleavage assay

In vitro cleavage of recombinant GST‐DLP1 protein by calpain was performed as previously described (Garg, Timm, Mandelkow, Mandelkow, & Wang, 2011). Briefly, recombinant GST‐DLP1 (160 ng; Abnova, Walnut, CA, USA) was incubated with calpain‐1 (Biovision, San Francisco, CA, USA) in reaction buffer for various times with or without calpeptin (50 μM; Tocris Bioscience, Minneapolis, MN, USA). After being incubated for the indicated times, the reaction mixture was mixed with an equal volume of 2× SDS sample buffer and boiled for 10 min. Samples were subjected to SDS‐PAGE followed by Western Blotting with anti‐DLP1 D6C7 (Cell Signaling Technology, Danvers, MA, USA) or anti‐GST (Santa Cruz, Dallas, TX, USA) antibodies.

### 4.5 Western Blotting

Samples of frozen cortical gray matter of AD (*N* = 7), and age‐ and gender‐matched control cases (*N* = 7) were homogenized and lysed with RIPA Buffer (Abcam) plus 1 mM phenylmethylsulfonyl fluoride (Sigma) and Protease Inhibitor Cocktail (Sigma) and centrifuged for 10 min at 16,000 × *g* at 4°C. Protein concentrations of the lysates from total cortical gray matter homogenates were determined by the bicinchoninic acid assay method (Pierce, Rockford, IL, USA). Equal amounts of proteins (20 μg) were separated by sodium dodecyl sulfate–polyacrylamide gel electrophoresis (SDS‐PAGE) and transferred to immobilon membranes. After blocking with 10% nonfat dry milk, primary and secondary antibodies were applied and the blots developed with enhanced chemiluminescence (Santa Cruz).

Cell lysates from primary neurons were prepared with protein extraction solution (Cell Signaling Technology) in accordance with the manufacturer's guidelines. Proteins were subjected to SDS‐PAGE and subsequently transferred to PVDF membrane (Bio‐Rad, Hercules, CA, USA) and blocked with 5% skim milk in TBST buffer. Blots were incubated for 16 hr at 4°C with primary antibodies to DLP1 D6C7 (1:1,000; Cell Signaling), calpain (1:2,000; Catalog#2556, Cell Signaling), spectrin (1:1,000; Cell Signaling), actin C4 (1:5,000; Thermo Fisher Scientific), DLP1 C‐5 (1:1,000; Santa Cruz), and GAPDH (1:2000; Cell Signaling). The blots were washed in TBST buffer, incubated with secondary antibodies for 1 hr at 23°C, and visualized using enhanced chemiluminescence reagents (Santa Cruz).

### 4.6 Immunocytochemical procedures

Hippocampus samples from AD (*n* = 6) and neuropathologically normal cases (*n* = 5) were fixed in formalin and paraffin embedded. Tissue sections 6 micron thick were used for immunostaining as described previously (Jiang et al., 2018). Antigen retrieval was performed in a pressure cooker using acetate buffer (Biocare Medical). Serial adjacent sections were immunostained for DLP1 and calpain‐1 with identical conditions for all cases used. Images of the CA2 neurons were obtained on a Zeiss Axioskop and densitometric analysis performed using Axiovision software to determine staining intensity.

### 4.7 Statistical analysis

Data are presented as means ± standard error of the mean (*SEM*) of at least three independent experiments and, where appropriate, were analyzed using one‐way analysis of variance (ANOVA) or Student's *t* test. *p* < 0.05 was considered statistically significant.

## CONFLICT OF INTEREST

None declared.

## AUTHOR CONTRIBUTIONS

X.Z. conceived and directed the project, analyzed/interpreted the results, and wrote the manuscript. S.J., C.S., F.T, and W.W. designed and carried out experiments, analyzed results, and generated figures. S.J. and W.W. drafted manuscript. All authors read and commented on manuscript drafts.

## References

[acel12912-bib-0001] Atherton, J. , Kurbatskaya, K. , Bondulich, M. , Croft, C. L. , Garwood, C. J. , Chhabra, R. , … Noble, W. (2014). Calpain cleavage and inactivation of the sodium calcium exchanger‐3 occur downstream of Abeta in Alzheimer's disease. Aging Cell, 13(1), 49–59. 10.1111/acel.12148 23919677 PMC4326873

[acel12912-bib-0002] Barsoum, M. J. , Yuan, H. , Gerencser, A. A. , Liot, G. , Kushnareva, Y. , Graber, S. , … Bossy‐Wetzel, E. (2006). Nitric oxide‐induced mitochondrial fission is regulated by dynamin‐related GTPases in neurons. EMBO Journal, 25(16), 3900–3911. 10.1038/sj.emboj.7601253 16874299 PMC1553198

[acel12912-bib-0003] Bossy, B. , Petrilli, A. , Klinglmayr, E. , Chen, J. , Lutz‐Meindl, U. , Knott, A. B. , … Bossy‐Wetzel, E. (2010). S‐Nitrosylation of DRP1 does not affect enzymatic activity and is not specific to Alzheimer's disease. Journal of Alzheimer's Disease, 20(Suppl 2), S513–S526. 10.3233/JAD-2010-100552 PMC2893334 20463395

[acel12912-bib-0004] Cereghetti, G. M. , Stangherlin, A. , Martins de Brito, O. , Chang, C. R. , Blackstone, C. , Bernardi, P. , & Scorrano, L. (2008). Dephosphorylation by calcineurin regulates translocation of Drp1 to mitochondria. Proceedings of the National Academy of Sciences of the United States of America, 105(41), 15803–15808. 10.1073/pnas.0808249105 18838687 PMC2572940

[acel12912-bib-0005] De Vos, K. J. , Allan, V. J. , Grierson, A. J. , & Sheetz, M. P. (2005). Mitochondrial function and actin regulate dynamin‐related protein 1‐dependent mitochondrial fission. Current Biology, 15(7), 678–683. 10.1016/j.cub.2005.02.064 15823542

[acel12912-bib-0006] Du, H. , Guo, L. , Yan, S. , Sosunov, A. A. , McKhann, G. M. , & Yan, S. S. (2010). Early deficits in synaptic mitochondria in an Alzheimer's disease mouse model. Proceedings of the National Academy of Sciences of the United States of America, 107(43), 18670–18675. 10.1073/pnas.1006586107 20937894 PMC2972922

[acel12912-bib-0007] Estaquier, J. , & Arnoult, D. (2007). Inhibiting Drp1‐mediated mitochondrial fission selectively prevents the release of cytochrome c during apoptosis. Cell Death and Differentiation, 14(6), 1086–1094. 10.1038/sj.cdd.4402107 17332775

[acel12912-bib-0008] Ferreira, A. , & Bigio, E. H. (2011). Calpain‐mediated tau cleavage: A mechanism leading to neurodegeneration shared by multiple tauopathies. Molecular Medicine, 17(7–8), 676–685. 10.2119/molmed.2010.00220 21442128 PMC3146621

[acel12912-bib-0009] Frohlich, C. , Grabiger, S. , Schwefel, D. , Faelber, K. , Rosenbaum, E. , Mears, J. , … Daumke, O. (2013). Structural insights into oligomerization and mitochondrial remodelling of dynamin 1‐like protein. EMBO Journal, 32(9), 1280–1292. 10.1038/emboj.2013.74 23584531 PMC3642683

[acel12912-bib-0010] Garg, S. , Timm, T. , Mandelkow, E. M. , Mandelkow, E. , & Wang, Y. (2011). Cleavage of Tau by calpain in Alzheimer's disease: The quest for the toxic 17 kD fragment. Neurobiology of Aging, 32(1), 1–14. 10.1016/j.neurobiolaging.2010.09.008 20961659

[acel12912-bib-0011] Hirai, K. , Aliev, G. , Nunomura, A. , Fujioka, H. , Russell, R. L. , Atwood, C. S. , … Smith, M. A. (2001). Mitochondrial abnormalities in Alzheimer's disease. Journal of Neuroscience, 21(9), 3017–3023. 10.1523/JNEUROSCI.21-09-03017.2001 11312286 PMC6762571

[acel12912-bib-0012] Jiang, S. , Nandy, P. , Wang, W. , Ma, X. , Hsia, J. , Wang, C. , … Zhu, X. (2018). Mfn2 ablation causes an oxidative stress response and eventual neuronal death in the hippocampus and cortex. Molecular Neurodegeneration, 13(1), 5 10.1186/s13024-018-0238-8 29391029 PMC5796581

[acel12912-bib-0013] Jin, N. , Yin, X. , Yu, D. , Cao, M. , Gong, C. X. , Iqbal, K. , … Liu, F. (2015). Truncation and activation of GSK‐3beta by calpain I: A molecular mechanism links to tau hyperphosphorylation in Alzheimer's disease. Scientific Reports, 5, 8187 10.1038/srep08187 25641096 PMC4313118

[acel12912-bib-0014] Kaech, S. , & Banker, G. (2006). Culturing hippocampal neurons. Nature Protocols, 1(5), 2406–2415. 10.1038/nprot.2006.356 17406484

[acel12912-bib-0015] Kamat, P. K. , Rai, S. , & Nath, C. (2013). Okadaic acid induced neurotoxicity: An emerging tool to study Alzheimer's disease pathology. Neurotoxicology, 37, 163–172. 10.1016/j.neuro.2013.05.002 23688530

[acel12912-bib-0016] Kandimalla, R. , & Reddy, P. H. (2016). Multiple faces of dynamin‐related protein 1 and its role in Alzheimer's disease pathogenesis. Biochimica Et Biophysica Acta, 1862(4), 814–828. 10.1016/j.bbadis.2015.12.018 26708942 PMC5343673

[acel12912-bib-0017] Kelly, B. L. , Vassar, R. , & Ferreira, A. (2005). Beta‐amyloid‐induced dynamin 1 depletion in hippocampal neurons. A potential mechanism for early cognitive decline in Alzheimer disease. Journal of Biological Chemistry, 280(36), 31746–31753. 10.1074/jbc.M503259200 16002400 PMC1364535

[acel12912-bib-0018] LaFerla, F. M. (2002). Calcium dyshomeostasis and intracellular signalling in Alzheimer's disease. Nature Reviews Neuroscience, 3(11), 862–872. 10.1038/nrn960 12415294

[acel12912-bib-0019] Li, S. , Jin, M. , Koeglsperger, T. , Shepardson, N. E. , Shankar, G. M. , & Selkoe, D. J. (2011). Soluble Abeta oligomers inhibit long‐term potentiation through a mechanism involving excessive activation of extrasynaptic NR2B‐containing NMDA receptors. Journal of Neuroscience, 31(18), 6627–6638. 10.1523/JNEUROSCI.0203-11.2011 21543591 PMC3100898

[acel12912-bib-0020] Li, Z. , Okamoto, K. , Hayashi, Y. , & Sheng, M. (2004). The importance of dendritic mitochondria in the morphogenesis and plasticity of spines and synapses. Cell, 119(6), 873–887. 10.1016/j.cell.2004.11.003 15607982

[acel12912-bib-0021] Liu, F. , Grundke‐Iqbal, I. , Iqbal, K. , Oda, Y. , Tomizawa, K. , & Gong, C. X. (2005). Truncation and activation of calcineurin A by calpain I in Alzheimer disease brain. Journal of Biological Chemistry, 280(45), 37755–37762. 10.1074/jbc.M507475200 16150694

[acel12912-bib-0022] Loucks, F. A. , Schroeder, E. K. , Zommer, A. E. , Hilger, S. , Kelsey, N. A. , Bouchard, R. J. , … Linseman, D. A. (2009). Caspases indirectly regulate cleavage of the mitochondrial fusion GTPase OPA1 in neurons undergoing apoptosis. Brain Research, 1250, 63–74. 10.1016/j.brainres.2008.10.081 19046944 PMC2771186

[acel12912-bib-0023] Manczak, M. , Calkins, M. J. , & Reddy, P. H. (2011). Impaired mitochondrial dynamics and abnormal interaction of amyloid beta with mitochondrial protein Drp1 in neurons from patients with Alzheimer's disease: Implications for neuronal damage. Human Molecular Genetics, 20(13), 2495–2509. 10.1093/hmg/ddr139 21459773 PMC3109997

[acel12912-bib-0024] Manczak, M. , & Reddy, P. H. (2012). Abnormal interaction between the mitochondrial fission protein Drp1 and hyperphosphorylated tau in Alzheimer's disease neurons: Implications for mitochondrial dysfunction and neuronal damage. Human Molecular Genetics, 21(11), 2538–2547. 10.1093/hmg/dds072 22367970 PMC3349426

[acel12912-bib-0025] Manczak, M. , Mao, P. , Calkins, M. J. , Cornea, A. , Reddy, A. P. , Murphy, M. P. , … Reddy, P. H. (2010). Mitochondria‐targeted antioxidants protect against amyloid‐beta toxicity in Alzheimer's disease neurons. Journal of Alzheimer's Disease, 20(Suppl 2), S609–S631. 10.3233/JAD-2010-100564 PMC3072711 20463406

[acel12912-bib-0026] Mattson, M. P. (2004). Pathways towards and away from Alzheimer's disease. Nature, 430(7000), 631–639. 15295589 10.1038/nature02621 PMC3091392

[acel12912-bib-0027] McKhann, G. , Drachman, D. , Folstein, M. , Katzman, R. , Price, D. , & Stadlan, E. M. (1984). Clinical diagnosis of Alzheimer's disease: Report of the NINCDS‐ADRDA Work Group under the auspices of Department of Health and Human Services task force on Alzheimer's disease. Neurology, 34(7), 939–944. 10.1212/WNL.34.7.939 6610841

[acel12912-bib-0028] Mears, J. A. , Lackner, L. L. , Fang, S. , Ingerman, E. , Nunnari, J. , & Hinshaw, J. E. (2011). Conformational changes in Dnm1 support a contractile mechanism for mitochondrial fission. Nature Structural & Molecular Biology, 18(1), 20–26. 10.1038/nsmb.1949 PMC3059246 21170049

[acel12912-bib-0029] Medeiros, R. , Kitazawa, M. , Chabrier, M. A. , Cheng, D. , Baglietto‐Vargas, D. , Kling, A. , … LaFerla, F. M. (2012). Calpain inhibitor A‐705253 mitigates Alzheimer's disease‐like pathology and cognitive decline in aged 3xTgAD mice. American Journal of Pathology, 181(2), 616–625. 10.1016/j.ajpath.2012.04.020 22688056

[acel12912-bib-0030] Mishra, P. , & Chan, D. C. (2014). Mitochondrial dynamics and inheritance during cell division, development and disease. Nature Reviews Molecular Cell Biology, 15(10), 634–646. 10.1038/nrm3877 25237825 PMC4250044

[acel12912-bib-0031] Morales‐Corraliza, J. , Berger, J. D. , Mazzella, M. J. , Veeranna , Neubert, T. A. , Ghiso, J. , … Mathews, P. M. (2012). Calpastatin modulates APP processing in the brains of beta‐amyloid depositing but not wild‐type mice. Neurobiology of Aging, 33(6), 1125e9–18. 10.1016/j.neurobiolaging.2011.11.023 PMC3318946 22206846

[acel12912-bib-0032] Otera, H. , Ishihara, N. , & Mihara, K. (2013). New insights into the function and regulation of mitochondrial fission. Biochimica Et Biophysica Acta, 1833(5), 1256–1268. 10.1016/j.bbamcr.2013.02.002 23434681

[acel12912-bib-0033] Reddy, P. H. , Reddy, T. P. , Manczak, M. , Calkins, M. J. , Shirendeb, U. , & Mao, P. (2011). Dynamin‐related protein 1 and mitochondrial fragmentation in neurodegenerative diseases. Brain Research Reviews, 67(1–2), 103–118. 10.1016/j.brainresrev.2010.11.004 21145355 PMC3061980

[acel12912-bib-0034] Saito, K. , Elce, J. S. , Hamos, J. E. , & Nixon, R. A. (1993). Widespread activation of calcium‐activated neutral proteinase (calpain) in the brain in Alzheimer disease: A potential molecular basis for neuronal degeneration. Proceedings of the National Academy of Sciences of the United States of America, 90(7), 2628–2632. 10.1073/pnas.90.7.2628 8464868 PMC46148

[acel12912-bib-0035] Sesaki, H. , Adachi, Y. , Kageyama, Y. , Itoh, K. , & Iijima, M. (2014). In vivo functions of Drp1: Lessons learned from yeast genetics and mouse knockouts. Biochimica Et Biophysica Acta, 1842(8), 1179–1185. 10.1016/j.bbadis.2013.11.024 24326103 PMC4048796

[acel12912-bib-0036] Simpkins, K. L. , Guttmann, R. P. , Dong, Y. , Chen, Z. , Sokol, S. , Neumar, R. W. , & Lynch, D. R. (2003). Selective activation induced cleavage of the NR2B subunit by calpain. Journal of Neuroscience, 23(36), 11322–11331. 10.1523/JNEUROSCI.23-36-11322.2003 14672996 PMC6740527

[acel12912-bib-0037] Song, H. L. , Shim, S. , Kim, D. H. , Won, S. H. , Joo, S. , Kim, S. , … Yoon, S. Y. (2014). beta‐Amyloid is transmitted via neuronal connections along axonal membranes. Annals of Neurology, 75(1), 88–97. 10.1002/ana.24029 24114864

[acel12912-bib-0038] Swerdlow, R. H. (2016). Bioenergetics and metabolism: A bench to bedside perspective. Journal of Neurochemistry, 139(Suppl 2), 126–135. 10.1111/jnc.13509 26968700 PMC5851778

[acel12912-bib-0039] Trushina, E. , Nemutlu, E. , Zhang, S. , Christensen, T. , Camp, J. , Mesa, J. , … Poduslo, J. F. (2012). Defects in mitochondrial dynamics and metabolomic signatures of evolving energetic stress in mouse models of familial Alzheimer's disease. PLoS ONE, 7(2), e32737 10.1371/journal.pone.0032737 22393443 PMC3290628

[acel12912-bib-0040] Wang, S. , Song, J. , Tan, M. , Albers, K. M. , & Jia, J. (2012). Mitochondrial fission proteins in peripheral blood lymphocytes are potential biomarkers for Alzheimer's disease. European Journal of Neurology, 19(7), 1015–1022. 10.1111/j.1468-1331.2012.03670.x 22340708

[acel12912-bib-0041] Wang, W. , Yin, J. , Ma, X. , Zhao, F. , Siedlak, S. L. , Wang, Z. , … Zhu, X. (2017). Inhibition of mitochondrial fragmentation protects against Alzheimer's disease in rodent model. Human Molecular Genetics, 26(21), 4118–4131. 10.1093/hmg/ddx299 28973308 PMC5886251

[acel12912-bib-0042] Wang, X. , Su, B. , Fujioka, H. , & Zhu, X. (2008). Dynamin‐like protein 1 reduction underlies mitochondrial morphology and distribution abnormalities in fibroblasts from sporadic Alzheimer's disease patients. American Journal of Pathology, 173(2), 470–482. 10.2353/ajpath.2008.071208 18599615 PMC2475784

[acel12912-bib-0043] Wang, X. , Su, B. , Lee, H. G. , Li, X. , Perry, G. , Smith, M. A. , & Zhu, X. (2009). Impaired balance of mitochondrial fission and fusion in Alzheimer's disease. Journal of Neuroscience, 29(28), 9090–9103. 10.1523/JNEUROSCI.1357-09.2009 19605646 PMC2735241

[acel12912-bib-0044] Wang, X. , Wang, W. , Li, L. , Perry, G. , Lee, H. G. , & Zhu, X. (2014). Oxidative stress and mitochondrial dysfunction in Alzheimer's disease. Biochimica Et Biophysica Acta, 1842(8), 1240–1247. 10.1016/j.bbadis.2013.10.015 24189435 PMC4007397

[acel12912-bib-0045] Wang, X. , Su, B. , Siedlak, S. L. , Moreira, P. I. , Fujioka, H. , Wang, Y. , … Zhu, X. (2008). Amyloid‐beta overproduction causes abnormal mitochondrial dynamics via differential modulation of mitochondrial fission/fusion proteins. Proceedings of the National Academy of Sciences of the United States of America, 105(49), 19318–19323. 10.1073/pnas.0804871105 19050078 PMC2614759

[acel12912-bib-0046] Xie, H. , Guan, J. , Borrelli, L. A. , Xu, J. , Serrano‐Pozo, A. , & Bacskai, B. J. (2013). Mitochondrial alterations near amyloid plaques in an Alzheimer's disease mouse model. Journal of Neuroscience, 33(43), 17042–17051. 10.1523/JNEUROSCI.1836-13.2013 24155308 PMC3807029

[acel12912-bib-0047] Xu, S. , Wang, P. , Zhang, H. , Gong, G. , Gutierrez Cortes, N. , Zhu, W. , … Wang, W. (2016). CaMKII induces permeability transition through Drp1 phosphorylation during chronic beta‐AR stimulation. Nature Communications, 7, 13189 10.1038/ncomms13189 PMC5067512 27739424

[acel12912-bib-0048] Yin, J. , Zhao, F. , Chojnacki, J. E. , Fulp, J. , Klein, W. L. , Zhang, S. , & Zhu, X. (2018). NLRP3 inflammasome inhibitor ameliorates amyloid pathology in a mouse model of Alzheimer's disease. Molecular Neurobiology, 55(3), 1977–1987. 10.1007/s12035-017-0467-9 28255908 PMC5585057

[acel12912-bib-0049] Zhao, F. , Wang, W. , Wang, C. , Siedlak, S. L. , Fujioka, H. , Tang, B. , & Zhu, X. (2017). Mfn2 protects dopaminergic neurons exposed to paraquat both in vitro and in vivo: Implications for idiopathic Parkinson's disease. Biochimica Et Biophysica Acta, 1863(6), 1359–1370. 10.1016/j.bbadis.2017.02.016 28215578 PMC5474135

