# Supplementary figures and images for: Dynamin‐like protein 1 cleavage by calpain in Alzheimer’s disease
Source: Aging Cell. 2019 Feb 14;18(3):e12912. doi: 10.1111/acel.12912 (PMC6516178; doi:10.1111/acel.12912)

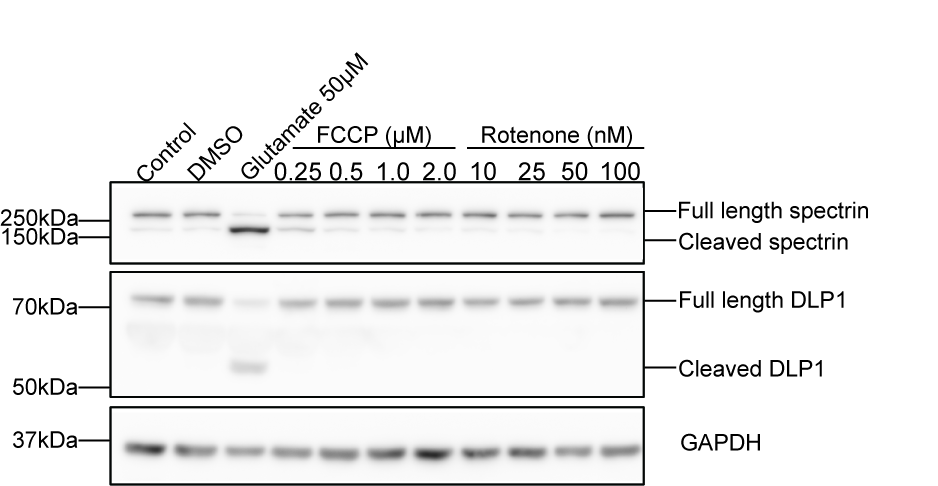

Supplement: Supplementary file 1 [file ACEL-18-e12912-s001.tif]
